# Interim effectiveness of trivalent influenza vaccine in a season dominated by lineage mismatched influenza B, northern Spain, 2017/18

**DOI:** 10.2807/1560-7917.ES.2018.23.7.18-00057

**Published:** 2018-02-15

**Authors:** Jesús Castilla, Ana Navascués, Itziar Casado, Alejandra Pérez-García, Aitziber Aguinaga, Guillermo Ezpeleta, Francisco Pozo, Carmen Ezpeleta, Iván Martínez-Baz, Primary Health Care Sentinel Network, Network for Influenza Surveillance in Hospitals of Navarre

**Affiliations:** 1 Instituto de Salud Pública de Navarra, IdiSNA - Navarre Institute for Health Research, Pamplona, Spain; 2 CIBER Epidemiología y Salud Pública (CIBERESP), Spain; 3 Servicio de Microbiología, Complejo Hospitalario de Navarra, IdiSNA - Navarre Institute for Health Research, Pamplona, Spain; 4 Centro Nacional de Microbiología (WHO National Influenza Centre - Madrid), Instituto de Salud Carlos III, Majadahonda, Spain; 5 The members of these networks are listed at the end of the article.

**Keywords:** influenza, influenza vaccine, influenza-like illness, case-control study, vaccine effectiveness, repeated vaccination

## Abstract

The 2017/18 interim estimate of trivalent influenza vaccine effectiveness (VE) was 39% (95% confidence interval: 20–54) in Navarre. Compared with individuals unvaccinated in the current and five previous seasons, VE against influenza B was 41% for current and any prior doses, 67% for current vaccination only, and 22% for any prior doses, and 43%, 51% and 54%, respectively against influenza A(H3N2). This suggests moderate VE despite predominance of lineage mismatched influenza B.

The early 2017/18 influenza season in Europe was characterised by co-circulation of influenza B, A(H3N2) and A(H1N1)pdm09, with lineage mismatched influenza B(Yamagata) virus predominating in many countries [1,2]. Concerns arose due to the low influenza vaccine effectiveness (VE) reported in the 2017 influenza A(H3N2) epidemic in Australia [3] and the warning about low VE of the trivalent influenza vaccine (TIV) against a lineage mismatched influenza B(Yamagata) virus [4]. Influenza vaccination in previous seasons may retain some preventive effect and modify the effect of the current season vaccination so the vaccination history should be considered in the VE assessment [5,6].

We present the 2017/18 interim effectiveness estimates of different combinations of current and prior season influenza vaccination in preventing laboratory-confirmed influenza.

## Study design and information sources

A test-negative case–control study was used for the estimations. Cases and controls were identified through the influenza epidemiological and virological surveillance in primary healthcare and hospitals in Navarre, northern Spain. In October and November 2017, the trivalent inactivated non-adjuvanted vaccine was offered free of charge to the target population for vaccination, which included people aged 60 years or more and people with major chronic conditions. The TIV comprised influenza A/Michigan/45/2015(H1N1)-like, A/HongKong/4801/2014(H3N2)-like and B/Brisbane/60/2008(Victoria-lineage)-like antigens [7]. The TIV had contained B(Yamagata) antigens in the 2012/13 to 2015/16 seasons [8]. Influenza vaccine status in the current and five prior influenza seasons, 2012/13 to 2017/18, was obtained from the regional vaccination register, where all vaccines administered in healthcare centres are registered online [9]. Persons were considered to be protected by the vaccine 14 days after receiving it.

Influenza surveillance relied on all primary healthcare physicians and hospitals automatically reporting influenza-like illness (ILI) cases [6]. A sentinel network of primary healthcare physicians collected nasopharyngeal and pharyngeal swabs from their patients diagnosed with ILI, when symptoms had appeared less than five days before. In hospitals, early detection and swabbing of all hospitalised patients with ILI was specified by the protocol. Samples were processed by reverse-transcription PCR assay. A selection of representative strains of each week and virus type/subtype was sent to the National Influenza Centre–Madrid laboratory to be completely genetically characterised.

## Statistical analysis

The study population included individuals covered by the Navarre Health Service since 2012 (96% of the population). All ILI patients who were swabbed in December 2017 and January 2018 were considered. We excluded healthcare workers, people living in nursing homes, children under 9 years of age and patients hospitalised prior to ILI symptom onset. The seasonal vaccination status of patients testing positive for influenza virus (cases) was compared to that of those who were negative for this virus (controls). Logistic regression models were employed to derive crude and adjusted odds ratios (OR) with their 95% confidence intervals (CI). Adjusted models included sex, age group (9–24, 25–44, 45–64, 65–84 and ≥ 85 years), major chronic conditions, month of swabbing and healthcare setting. Four categories combining the current-season and five prior season vaccination were considered: current-season vaccination and any prior doses, current-season vaccination and no prior doses, no current-season vaccination and any prior doses, and no current-season vaccination and no prior doses (reference group). VE was estimated as a percentage: (1 – OR) x 100.

## Influenza vaccine effectiveness interim estimation

A total of 1,268 ILI patients were included, 808 (64%) inpatients and 460 (36%) primary healthcare patients. A total of 654 (52%) were confirmed cases for influenza virus: 498 (76%) for influenza B, 118 (18%) for A(H3N2), 36 (6%) for A(H1N1)pdm09 and two non-subtyped influenza A viruses.

The sequence derived from the amplification product of the HA1 fragment of the haemagglutinin gene was characterised for 51 viruses. Of 40 influenza B viruses, 35 were B/Phuket/3073/2013(Yamagata-lineage)-like, three B/Brisbane/60/2008(Victoria-lineage)-like and two B/Norway/2409/2017(Victoria-lineage)-like. The four A(H1N1)pdm09 strains were A/Michigan/45/2015-like. Among seven A(H3N2) strains, five were A/HongKong/4801/2014-like and two A/Singapore/16–0019/2016-like.

Compared with test-negative controls, influenza cases comprised a lower proportion of individuals aged 65 years or older, of persons with comorbidities or who were attended in hospitals. Among cases, 35% (231/654) had been vaccinated in the 2017/18 season vs 54% (331/614) among controls (p < 0.001) (Table 1).

**Table 1:** Characteristics of the patients with medically-attended influenza-like illness included in the test-negative case–control analysis, Navarre, Spain, December 2017–January 2018 (n = 1,268 patients)

Characteristics	Test-negative controls		All influenza cases		Influenza B		Influenza A(H3N2)		Influenza A(H1N1)pdm09	
	N	%	N	%	N	%	N	%	N	%
**Age groups (years)**										
9–24	14	2	35	5	30	6	2	2	3	8
25–44	89	15	150	23	109	22	27	22	14	39
45–64	123	20	170	26	136	27	25	21	8	22
65–84	271	44	203	31	158	32	37	32	8	22
≥ 85	117	19	96	15	65	13	27	23	3	8
**Sex**										
Male	336	55	311	48	230	46	62	52	18	50
Female	278	45	343	52	268	54	56	48	18	50
**Major chronic conditions**										
No	167	27	278	43	221	44	37	31	19	53
Yes	447	73	376	57	277	56	80	69	17	47
**Month of swabbing**										
December	178	29	91	14	76	15	11	9	4	11
January	436	71	563	86	422	85	107	91	32	89
**Target group for vaccination^a^**										
No	110	18	210	32	164	33	27	23	18	50
Yes	504	82	444	68	334	67	91	77	18	50
**Healthcare setting**										
Primary healthcare	131	21	329	50	264	53	43	37	22	61
Hospitalization	483	79	325	50	234	47	75	63	14	39
**2017/18 season vaccine**										
No	283	46	423	65	328	66	66	56	28	78
Yes	331	54	231	35	170	34	52	44	8	22
**Total**	**614**	**100**	**654**	**100^b^**	**498**	**100**	**118**	**100**	**36**	**100**

^a^ Target group for vaccination includes people ≥ 60 years-old and people with major chronic conditions.

^b^ Two cases were influenza A not subtyped.

Regardless of the vaccination history, the overall adjusted estimate of influenza VE was 39% (95% CI: 20 to 54). In persons less than 65 years-old the estimates were higher (55%) than in the older age group (30%), and in outpatients (51%) than inpatients (35%). VE was 41% (95% CI: 20 to 56) against influenza B, 29% (95% CI: –15 to 57) against A(H3N2), and 59% (95% CI: –6 to 84) against A(H1N1)pdm09 (Table 2).

**Table 2:** Influenza vaccine effectiveness in preventing laboratory-confirmed influenza among individuals aged 9 years or older in Navarre, Spain, December 2017–January 2018 (n = 1,268 patients)

Models	Controls Vaccinated/unvaccinated	Cases Vaccinated/unvaccinated	Crude vaccine effectiveness % (95% CI)	Adjusted vaccine effectiveness % (95% CI)^a^
**All swabbed patients**	331/283	231/423	53 (42 to 63)	39 (20 to 54)
**Target group for vaccination^b^**	318/186	216/228	45 (28 to 57)	39 (17 to 54)
**Age group**				
9–64 years	54/172	41/314	58 (35 to 73)	55 (26 to 73)
≥ 65 years	277/111	190/109	30 (4 to 49)	30 (2 to 50)
**Virus type/subtype**				
Influenza B	331/283	170/328	56 (43 to 65)	41 (20 to 56)
Influenza A(H3N2)	331/283	52/66	33 (0 to 55)	29 (–15 to 57)
Influenza A(H1N1)pdm09	331/283	8/28	76 (46 to 89)	59 (–6 to 84)
**Primary healthcare patients**				
All influenza viruses	35/96	56/273	44 (9 to 65)	51 (13 to 73)
Influenza B	35/96	47/217	41 (2 to 64)	52 (12 to 74)
Influenza A(H3N2)	35/96	6/37	56 (–14 to 83)	54 (–44 to 85)
Influenza A(H1N1)pdm09	35/96	3/19	57 (–55 to 88)	49 (–120 to 88)
**Hospitalised patients**				
All influenza viruses	296/187	175/150	26 (2 to 45)	35 (11 to 53)
Influenza B	296/187	123/111	30 (4 to 49)	37 (11 to 55)
Influenza A(H3N2)	296/187	46/29	0 (–65 to 39)	20 (–40 to 54)
Influenza A(H1N1)pdm09	296/187	5/9	65 (–6 to 88)	63 (–27 to 89)

CI: confidence interval.

**^a^** Logistic regression model adjusted for sex, age group (9–24, 25–44, 45–64, 65–85 and ≥ 85 years), major chronic conditions, month of swabbing and healthcare setting (primary healthcare and hospital).

^b^ Target group for vaccination includes people ≥ 60 years old and people with major chronic conditions.

Nevertheless, better levels of protection were observed in the analysis considering the vaccination history. Compared with persons never vaccinated in the current and five previous seasons, the preventive effect was 42% (95% CI: 20 to 58) in those vaccinated in the current and any prior seasons, 65% (95% CI: 32 to 82) in those vaccinated only in the current season, and 28% (95% CI: –11 to 53) in those vaccinated only in any prior seasons. The corresponding estimates against influenza B were 41% (95% CI: 17 to 59), 67% (95% CI: 31 to 84) and 22% (95% CI: –24 to 51), and against A(H3N2) were 43% (95% CI: –1 to 67), 51% (95% CI: –51 to 84) and 54% (95% CI: –7 to 80), respectively (Table 3).

**Table 3:** Effectiveness of current season influenza vaccination and of vaccination in the five prior seasons in preventing laboratory-confirmed influenza cases among people aged 9 years or older, Navarre, Spain, December 2017–January 2018 (n = 1,268 patients)

Vaccination history by type of patients or influenza	Cases/controls	Crude vaccine effectiveness % (95% CI)	Adjusted vaccine effectiveness % (95% CI)^a^
**All patients**			
Never vaccinated	366/211	Reference	Reference
No current + any prior dose	57/72	54 (33 to 69)	28 (–11 to 53)
Current only	17/28	65 (35 to 81)	65 (32 to 82)
Current + any prior dose	214/303	59 (48 to 68)	42 (20 to 58)
**Primary healthcare patients**			
Never vaccinated	261/87	Reference	Reference
No current + any prior dose	12/9	56 (–9 to 82)	51 (–25 to 81)
Current only	8/11	76 (38 to 91)	79 (42 to 92)
Current + any prior dose	48/24	33 (–15 to 61)	39 (–20 to 69)
**Hospitalised patients**			
Never vaccinated	105/124	Reference	Reference
No current + any prior dose	45/63	16 (–34 to 47)	20 (–31 to 52)
Current only	9/17	38 (–46 to 73)	47 (–28 to 78)
Current + any prior dose	166/279	30 (3 to 49)	41 (13 to 59)
**Influenza B**			
Never vaccinated	283/211	Reference	Reference
No current + any prior dose	45/72	53 (30 to 69)	22 (–24 to 51)
Current only	13/28	65 (32 to 83)	67 (31 to 84)
Current + any prior dose	157/303	61 (50 to 70)	41 (17 to 59)
**Influenza A(H3N2)**			
Never vaccinated	28/211	Reference	Reference
No current + any prior dose	8/72	60 (11 to 82)	54 (–7 to 80)
Current only	4/28	48 (–54 to 83)	51 (–51 to 84)
Current + any prior dose	48/303	42 (11 to 82)	43 (–1 to 67)

CI: confidence interval.

^a^ Vaccine effectiveness adjusted by age groups (9–24, 25–44, 45–64, 65–84 and ≥ 85 years), sex, major chronic conditions, healthcare setting (primary healthcare and hospital), and month of swabbing.

## Discussion

These results suggest a protective effect of the TIV of 42% to 65% in the early 2017/18 season in Navarre, depending on the vaccination status in prior seasons. Moderate VE was observed against influenza B, A(H1N1)pdm09 and A(H3N2).

Our results on influenza B are consistent with those recently reported from Canada [10] and contrast with the low VE expected in a season dominated by lineage mismatched influenza B virus [4]. Although we observed some preventive effect of previous vaccinations in individuals unvaccinated in the current season, the highest VE against influenza B was seen in people vaccinated in the current season but not vaccinated in prior ones, ruling out the possibility that the observed VE is due to the residual effect of previous vaccines containing B(Yamagata). Instead, this notable effectiveness of the TIV against influenza B suggests important cross-lineage protection [10-15].

The moderate VE against influenza A(H3N2) observed in the analysis adjusted for vaccination history contrasts with the lower estimate from the analysis that only considers current season vaccination, indicating that the vaccination history may be a confounding factor [6]. By including in the analyses any vaccination in the five prior seasons, the reference category was not affected by residual vaccine effect.

Our results from two independent groups, i.e. hospitalised patients and primary healthcare patients, were broadly consistent. The lower point estimates among inpatients in some analyses might be explained by the poorer immune response of patients who required hospitalisation.

This study has some limitations. The number of influenza B cases with known lineage was too small to obtain estimates by lineage, although 88% of known lineages were Yamagata. The results are preliminary and for some analyses, the statistical power is limited. Nevertheless, selection bias was reduced by recruiting laboratory-confirmed cases and controls in the same settings before either patient or physician was aware of laboratory results [16]. We also included outpatients and inpatients, thus obtaining broad representation of patients with influenza. The analyses were adjusted for the healthcare setting as this variable could have acted as a confounding factor.

In conclusion, these results suggest moderate effectiveness of the trivalent inactivated influenza vaccine against the three circulating viruses in the early 2017/18 season in northern Spain. The TIV effectiveness against influenza B suggests an important cross-lineage protection.

## Notes

**Members of the Primary Health Care Sentinel Network and of the Network for Influenza Surveillance in Hospitals of Navarre:** The members of the Primary Health Care Sentinel Network of Navarre are: I Abad, P Aldaz, R Ansorena, I Arcéiz, E Arina, MA Arrechea, I Arribas, N Aznárez, FC Bartolomé, A Beguiristain, A Beltrán, M Bidarte, I Blanco, A Brugos, B Cano, MV Castresana, JC Cenoz, F Cia, B Compains, JR Corpas, F Cortes, B Churío, PC Cuevas, EM Da Costa, MR Díez, FJ Escribano, M Esparza, MJ Esparza, V Etayo, C Fernández Alfaro, B Flamarique, ML Garcés, T García Albéniz, FJ García Nicolás, AB Germán, A Giner, JO Guiu, JC Gurbindo, MJ Guruchaga, JA Heras, M Hernández Galindo, MC Hijos, B Iñigo, MC Irigoyen, JJ Jurio, S Lizarraga, MJ Lizaso, JJ Longás, MJ López, MT Maquirriain, M Mazquiarán, JJ Miner, T Molins, M Moreno, MA Moros, U Navarro, E Orbara, M Orte, M Oscoz, P Palacio, J Palau, C Pérez Lecumberri, P Pérez Pascual, B Pérez Sanz, MJ Plumbed, A Prado Virto, M Prado Santamaría, A Puig Arrastia, M Ramos, BE Rípodas, M Rodríguez, MA Roncal, I Ruiz Puertas, C Sánchez, F Sánchez Miramón, P Sarrasqueta, F Satrústegui, MA Senosiáin, M Sota, ME Ursua, IA Urtasun, M Zardoya, ME Zubieta, F Elía, E Albéniz. The members of the Network for Influenza Surveillance in Hospitals of Navarre are: A Aguinaga, P Artajo, C Beaumont, X Beristain, J Chamorro, C Ezpeleta, F Gallinas, A Pérez-García, M Herranz, J Hueto, L Moreno, S Martínez-Pérez, A Navascués, N Viguria, J Sesma (Complejo Hospitalario de Navarra), I Estévez, JJ García Irure (Hospital Reina Sofía, Tudela), F Lameiro, AI Álvaro (Hospital García Orcoyen, Estella), I Casado, G Ezpeleta, M García Cenoz, J Castilla (Instituto de Salud Pública de Navarra).

## References

[1] European Centre for Disease Prevention and Control (ECDC). Weekly influenza update, week 5, January 2018. Stockholm: ECDC. [Accessed 12 Feb 2018]. Available from: https://ecdc.europa.eu/en/publications-data/weekly-influenza-update-week-5-january-2018

[2] Sistema de Vigilancia de la Gripe en España. Informe semanal de Vigilancia de la Gripe en España. Semana 5/2018 N° 531. [Weekly report of the influenza surveillance system in Spain 5/2018. No 531]. Madrid: Instituto de Salud Carlos III; 8 February 2018. Spanish. Available from: http://vgripe.isciii.es/documentos/20172018/boletines/grn052018.pdf

[3] Sullivan SG Chilver MB Carville KS Deng YM Grant KA Higgins G Low interim influenza vaccine effectiveness, Australia, 1 May to 24 September 2017. Euro Surveill. 2017;22(43):17-00707. 10.2807/1560-7917.ES.2017.22.43.17-00707 29090681 PMC5718387

[4] Wise J Trivalent flu vaccine won’t protect against influenza B strain predominantly circulating. BMJ. 2018;360:k78. 10.1136/bmj.k78 29305384

[5] McLean HQ Thompson MG Sundaram ME Meece JK McClure DL Friedrich TC Impact of repeated vaccination on vaccine effectiveness against influenza A(H3N2) and B during 8 seasons. Clin Infect Dis. 2014;59(10):1375-85. 10.1093/cid/ciu680 25270645 PMC4207422

[6] Castilla J Navascués A Casado I Díaz-González J Pérez-García A Fernandino L Primary Health Care Sentinel Network And The Network For Influenza Surveillance In Hospitals Of Navarre Combined effectiveness of prior and current season influenza vaccination in northern Spain: 2016/17 mid-season analysis. Euro Surveill. 2017;22(7):30465. 10.2807/1560-7917.ES.2017.22.7.30465 28230523 PMC5322189

[7] Recommended composition of influenza virus vaccines for use in the 2017–2018 northern hemisphere influenza season. Wkly Epidemiol Rec. 2017;92(11):117-28. 28303704

[8] Recommended composition of influenza virus vaccines for use in the 2016–2017 northern hemisphere influenza season. Wkly Epidemiol Rec. 2016;91(10):121-32. 26971356

[9] Aguilar I Reyes M Martínez-Baz I Guevara M Albéniz E Belza M Use of the vaccination register to evaluate influenza vaccine coverage in seniors in the 2010/11 influenza season, Navarre, Spain. Euro Surveill. 2012;17(17):20154. 10.2807/ese.17.17.20154-en 22551499

[10] Skowronski DM Chambers C De Serres G Dickinson JA Winter AL Hickman R Early season co-circulation of influenza A(H3N2) and B(Yamagata): interim estimates of 2017/18 vaccine effectiveness, Canada, January 2018. Euro Surveill. 2018;23(5):1800035. 10.2807/1560-7917.ES.2018.23.5.18-00035 29409570 PMC5801641

[11] McLean HQ Thompson MG Sundaram ME Kieke BA Gaglani M Murthy K Influenza vaccine effectiveness in the United States during 2012-2013: variable protection by age and virus type. J Infect Dis. 2015;211(10):1529-40. 10.1093/infdis/jiu647 25406334 PMC4407759

[12] Skowronski DM Janjua NZ Sabaiduc S De Serres G Winter AL Gubbay JB Influenza A/subtype and B/lineage effectiveness estimates for the 2011-2012 trivalent vaccine: cross-season and cross-lineage protection with unchanged vaccine. J Infect Dis. 2014;210(1):126-37. 10.1093/infdis/jiu048 24446529

[13] Janjua NZ Skowronski DM De Serres G Dickinson J Crowcroft NS Taylor M Estimates of influenza vaccine effectiveness for 2007-2008 from Canada’s sentinel surveillance system: cross-protection against major and minor variants. J Infect Dis. 2012;205(12):1858-68. 10.1093/infdis/jis283 22492921

[14] Skowronski DM Masaro C Kwindt TL Mak A Petric M Li Y Estimating vaccine effectiveness against laboratory-confirmed influenza using a sentinel physician network: results from the 2005-2006 season of dual A and B vaccine mismatch in Canada. Vaccine. 2007;25(15):2842-51. 10.1016/j.vaccine.2006.10.002 17081662

[15] Skowronski DM Chambers C Sabaiduc S De Serres G Winter AL Dickinson JA Beyond antigenic match: possible agent-host and immuno-epidemiological influences on influenza vaccine effectiveness during the 2015-2016 season in Canada. J Infect Dis. 2017;216(12):1487-500. 10.1093/infdis/jix526 29029166 PMC5853508

[16] Valenciano M Kissling E Ciancio BC Moren A Study designs for timely estimation of influenza vaccine effectiveness using European sentinel practitioner networks. Vaccine. 2010;28(46):7381-8. 10.1016/j.vaccine.2010.09.010 20851086

